# Detection of EGFR Mutations in cfDNA and CTCs, and Comparison to Tumor Tissue in Non-Small-Cell-Lung-Cancer (NSCLC) Patients

**DOI:** 10.3389/fonc.2020.572895

**Published:** 2020-10-08

**Authors:** Haiyan E. Liu, Meghah Vuppalapaty, Charles Wilkerson, Corinne Renier, Michael Chiu, Clementine Lemaire, James Che, Melissa Matsumoto, James Carroll, Steve Crouse, Violet R. Hanft, Stefanie S. Jeffrey, Dino Di Carlo, Edward B. Garon, Jonathan Goldman, Elodie Sollier

**Affiliations:** ^1^Vortex Biosciences, Inc., Pleasanton, CA, United States; ^2^Department of Bioengineering, University of California, Los Angeles, Los Angeles, CA, United States; ^3^Department of Medicine, David Geffen School of Medicine at UCLA, Los Angeles, CA, United States; ^4^Department of Surgery, Stanford University School of Medicine, Stanford, CA, United States; ^5^California NanoSystems Institute, Los Angeles, CA, United States; ^6^UCLA Jonsson Comprehensive Cancer Center, Los Angeles, CA, United States

**Keywords:** Vortex technology, circulating tumor cell, total liquid biopsy, epidermal grow factor receptor, EGFR mutation analysis, Non-small cell carcinoma, circulating tumor biomarkers, circulating free DNA (cfDNA)

## Abstract

Lung cancer is the leading cause of cancer-related mortality worldwide. Epidermal growth factor receptor (EGFR) tyrosine kinase inhibitor (TKI) therapies, based on the evaluation of *EGFR* mutations, have shown dramatic clinical benefits. *EGFR* mutation assays are mainly performed on tumor biopsies, which carry risks, are not always successful and give results relevant to the timepoint of the assay. To detect secondary *EGFR* mutations, which cause resistance to 1st and 2nd generation TKIs and lead to the administration of a 3rd generation drug, effective and non-invasive monitoring of *EGFR* mutation status is needed. Liquid biopsy analytes, such as circulating tumor cells (CTCs) and circulating tumor DNA (cfDNA), allow such monitoring over the course of the therapy. The aim of this study was to develop and optimize a workflow for the evaluation of cfDNA and CTCs in NSCLC patients all from one blood sample. Using Vortex technology and EntroGen ctEGFR assay, *EGFR* mutations were identified at 0.5 ng of DNA (∼83 cells), with a sensitivity ranging from 0.1 to 2.0% for a total DNA varying from 25 ng (∼4 CTCs among 4000 white blood cells, WBCs) to 1 ng (∼4 CTCs among 200 WBCs). The processing of plasma-depleted-blood provided comparable capture recovery as whole blood, confirming the possibility of a multimodality liquid biopsy analysis (cfDNA and CTC DNA) from a single tube of blood. Different anticoagulants were evaluated and compared in terms of respective performance. Blood samples from 24 NSCLC patients and 6 age-matched healthy donors were analyzed with this combined workflow to minimize blood volume needed and sample-to-sample bias, and the *EGFR* mutation profile detected from CTCs and cfDNA was compared to matched tumor tissues. Despite the limited size of the patient cohort, results from this non-invasive *EGFR* mutation analysis are encouraging and this combined workflow represents a valuable means for informing therapy selection and for monitoring treatment of patients with NSCLC.

## Introduction

Lung cancer is the leading cause of cancer deaths in the United States, among both men and women. An estimated 135,270 deaths from lung cancer occurred in 2019 (1). Non-Small Cell Lung Cancers (NSCLC), which include adenocarcinoma, squamous cell carcinoma, large cell carcinoma and large cell neuroendocrine tumors, account for ∼85% of primary lung cancers (2). Most NSCLC patients present with advanced or metastatic disease at diagnosis. With recent evidence showing that 10–30% of the NSCLC patients present “actionable” mutations of Epidermal Growth Factor Receptor (*EGFR*) (3), tremendous advances have been made in the treatment of these patients in recent years, by directly targeting these specific mutations (4).

**FIGURE 1:**
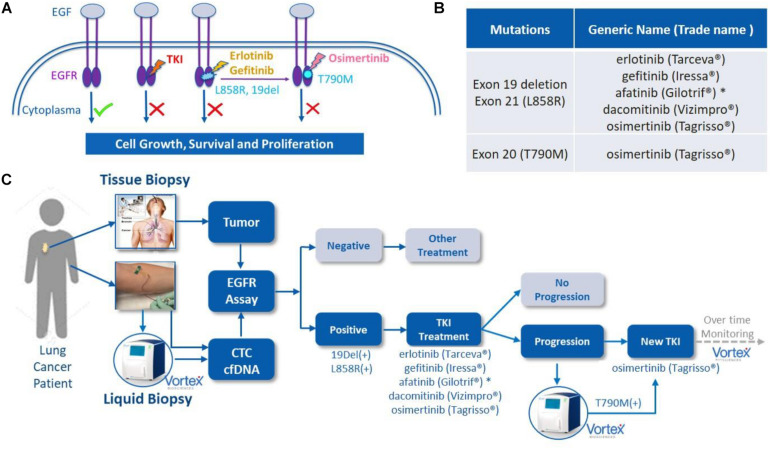
*EGFR* mutations, *EGFR* tyrosine kinase inhibitor (TKI) treatment and liquid biopsies. **(A)** EGF/*EGFR*, *EGFR* mutations and TKIs. When EGF binds to the extracellular binding domain of *EGFR*, the intracellular tyrosine kinase triggers several signal transduction cascades to regulate the cellular growth and proliferation. This effect can be blocked by TKIs, such as afatinib or erlotinib among others, when mutations of 19 Del or L858R are present in this kinase domain. A resistance often occurs due to the development of T790M mutation, which can then be blocked by second line TKI osimertinib. Original figure. **(B)** List of currently FDA approved TKIs. **(C)** The potential role of VTX-1 Liquid Biopsy System in the monitoring and treatment of lung cancer. By providing CTCs with high recovery, high purity, and with a simple and fully automated process, the VTX-1 enables an ongoing and non-invasive monitoring of *EGFR* mutations throughout the disease. * afatinib is approved in some rarer *EGFR* mutations as well. The pictures of Vortex instrument and the blood draw have been provided by Vortex Biosciences with the appropriate permissions. The picture of the thoracic biopsy has been revised from https://www.semanticscholar.org/paper/Chapter-2-Review-on-Image-Guided- Lung-Biopsy-Rizqie-Yusof/41e9a8569c1a3c89d4331f766215e4d8299e34cd.

Following the approval of the first *EGFR* tyrosine kinase inhibitor (TKI) gefitinib in 2003, other TKIs such as afatinib, erlotinib, dacomitinib, and osimertinib have been approved, from which many patients have benefited (Figure 1). Some of these drugs are now used as the first-line therapy for specific advanced NSCLCs. *EGFR* is a cellular transmembrane glycoprotein, consisting of (i) an extracellular epidermal growth factor (EGF)-binding-domain, and (ii) an intracellular tyrosine kinase domain, which when activated, triggers several signal transduction cascades that ultimately control cellular growth and proliferation (5). Several studies have shown that distinct mutation patterns in *EGFR*, including exon 19 deletion and exon 21 L858R substitution, were associated with positive treatment outcomes following first-line TKI therapy with afatinib, erlotinib, gefitinib and dacomitinib. These drugs can be administrated alone or, for a better overall survival, in combination with other treatment options. For example, the RELAY trial has demonstrated that a dual blockade of the *EGFR* and VEGF pathways (i.e., ramucirumab plus erlotinib) in *EGFR*-mutated untreated metastatic NSCLC patients provided superior progression-free survival compared to blocking the *EGFR* pathway only (placebo plus erlotinib) (6). In other studies, *EGFR* combined with cytotoxic chemotherapy (gefitinib plus carboplatin and pemetrexed) was identified as more effective than the *EGFR* TKI alone (7, 8). However, most patients eventually develop *EGFR*-TKI acquired resistance after a few months, due, partly, to the emergence of a new mutation known as *EGFR* T790M. Osimertinib (Tagrisso) is a second-line TKI that has demonstrated selectivity for T790M-resistant mutation in patients with advanced NSCLC and disease progression after prior *EGFR*-TKI therapy (Figure 1). Osimertinib can even be used as a frontline TKI, as demonstrated by the FLAURA trial (9). Therefore, the monitoring of these actionable mutations throughout the patient care is fundamental for the selection of suitable TKI treatment.

For most of clinical cases, tumor biopsies are used to evaluate the *EGFR* mutations and guide the patient treatment. However, lung tissue biopsies through needle biopsy or bronchoscopies may be painful, expensive, potentially risky for the patients, and not always successful (10). Sometimes, mutation profiling can be inaccurate due to inter or intra-tumoral heterogeneity. In some cases, re-biopsy may be required to obtain additional molecular information on the patient tumor. For monitoring the mutations over the course of the treatment, some patients with recurrence and poor overall status are not fit enough to have multiple biopsies. Thus, the capture of tumoral genomic content in bodily fluids such as blood, has been evaluated as an alternative for tissue biopsy (11). These so-called “liquid biopsies,” i.e., cell-free circulating tumor DNA (cfDNA) and circulating tumor cells (CTCs) are the main sources of tumor genomic material present in the blood and have been investigated for non-invasive detection and monitoring of tumors (12). Both liquid biopsies provide non-invasive ways to repeatedly sample cancer tumor(s) and set-up an *EGFR* mutation profiling to adjust treatment as the cancer evolves. Multiple studies have also reported the use of cfDNA or CTCs to assess the prognosis and to guide the treatment of NSCLC patients (13–15). Altogether, non-invasive liquid biopsies enable an ongoing evaluation of the NSCLC (16), to assess the cancer spread, to monitor the effects of the treatment (17, 18) and forewarn the physicians for possible recurrences (19), while also providing clues on the reasons for treatment inefficiency and cancer resistance (10).

VTX-1 Liquid Biopsy System (Vortex Biosciences) is a microfluidic label-free CTC isolation system, capturing CTCs based on their physical characteristics such as size and deformability instead of their surface markers (20–22). VTX-1 provides intact CTCs with high recovery and purity, alongside with a simple and fully automated process. This technology was described elsewhere in detail (20) and applied to various cancer types such as metastatic colorectal, breast, prostate and non-small-cell lung cancer (23–26). The blood sample can be depleted of its plasma first and further processed with the VTX-1 to recover the CTCs, thereby enabling the analysis of *EGFR* gene mutations in both CTCs and cfDNA from a single tube of blood.

The purpose of this study was to develop and characterize an integrated workflow for the profiling of *EGFR* mutations in NSCLC patients from cfDNA and CTCs from a single tube of blood. Different blood collection tubes were assessed in view of blood sample transportation and shipping to guarantee an optimal CTC recovery and DNA preservation. Finally, this workflow was applied to lung cancer patient blood samples as a preliminary validation. Ultimately, we compared the results of this combined liquid biopsy to the tumor tissue biopsy when available, in order to further investigate the feasibility of using non-invasive *EGFR* mutation analysis as a potential tool for monitoring treatment and medication guidance of NSCLC patients (Figure 1).

## Materials and Methods

### Cell Lines

Human NSCLC cell lines A549 (ATCC^®^ CCL-185^TM^, *EGFR* wild type), H1975 (ATCC^®^ CRL-5908^TM^, *EGFR* exon 20 T790M, and L858R exon 21 L858R mutations) and HCC827 (ATCC^®^ CRL-2868^TM^, *EGFR* exon 19 deletion), as well as MCF7 breast cancer cell line (ATCC^®^ HTB22^TM^) were used in this study. The cells were grown at 37°C and 5% CO2, in RPMI 1640 (H1975 and HCC827 cells), F-12K (A549 cells) or RPMI1640 + GlutaMax (MCF7 cells) medium (Gibco^®^), respectively, supplemented with 10% fetal bovine serum (HyClone) and 1% Penicillin-Streptomycin (Corning) and 0.01 mg/ml Human Recombinant Insulin for MCF7 cells.

Direct Sanger Sequencing was performed to confirm the mutation status of each lung cancer cell line (Supplementary Figure 1). To do so, DNA was extracted from the cells using the QIAamp DNA Micro Kit (Qiagen). The extracted DNA was quantified by Qubit Fluorometer (Thermo Fisher Scientific) and then subjected to PCR directly using primers against the *EGFR* exons 19, 20, and 21, covering hotspot regions of 19 deletion, T790M and L858R mutations (Supplementary Figure 2). After a control step with E-Gel Electrophoresis (Thermo Fisher Scientific) and Qubit for both PCR specificity and PCR product quantity, the PCR products were purified using the QIAquick PCR Purification Kit (Qiagen) and Sanger sequenced on a 3730XL DNA Analyzer (Elim Biopharmaceuticals). ABI chromatogram files were analyzed using the BioEdit sequence alignment editor.

### Donor Recruitment and Blood Collection

*(i) For assay development and characterization using spiked cell lines*, healthy volunteers were recruited according to a protocol with informed consent, as approved by the Institutional Review Board (protocol #5630) from Stanford University School of Medicine or through the Stanford Blood Center. Depending on the experiment, peripheral blood was collected into different blood collection tubes (BCTs): EDTA tube (BD Vacutainer^®^), CellSave Preservation Tubes (Janssen diagnostics LLC.), Streck Cell-Free DNA (Streck Inc.) or LBgard (Biomatrica). Immediately after the draw, the blood tubes were gently inverted 10 times, transported at room temperature (RT), and processed with Vortex technology.

*(ii) For EGFR assay validation with patient samples*, blood samples from 24 NSCLC patients and six age-matched healthy donors were recruited at David Geffen School of Medicine, University of California, Los Angeles (UCLA), according to the clinical study protocol UCLA IRB #11-001798. Before being enrolled into the study, all donors provided a written informed consent. All blood samples were deidentified to the persons doing the blood processing and *EGFR* assays. Donors’ age, diagnosis, disease stage and treatment history are summarized in the Table 1. Blood was collected in LBgard tubes, gently inverted 10 times immediately after the draw, and shipped in an insulated box (Saf-T-Pak #STP-302) with gel packs at room temperature (RT) from UCLA to Vortex headquarters, where they were processed within 1 h of reception.

**TABLE 1:** Clinical samples information.

Donor ID	Age	Histology	Stage	Treatment (years)
PA01	60–65	Adenocarcinoma	IV	afatinib (2016); erlotinib (2017 to time of blood collection)
PA02	75–80	Adenocarcinoma	IIIB	erlotinib (2010; 2014; 2016)
PA03.1	55–60	Adenocarcinoma	IV	Anti-PD1 (2016); Chemo (2017)
PA03.2				
PA03.3				
PA04.1	30–35	Squamous	IV	Anti-PD1 (2017)
PA04.2				
PA05.1	70–75	Adenocarcinoma	IV	Anti-PD1 and thoracentesis (2016)
PA05.2				
PA06.1	60–65	Adenocarcinoma	IV	erlotinib (2015); osimertinib (2016)
PA06.2				
PA07	65–70	Adenocarcinoma	IV	N/A
PA08	55–60	Adenocarcinoma	IA	erlotinib (2011–2015); rociletinib (2015); osimertinib (2015–2017); Chemo (2017)
PA09	60–65	Adenocarcinoma	IV	No treatment started at the time of blood collection
PA10	90–95	Adenocarcinoma	IV	No treatment started at the time of blood collection
PA11	55–60	Adenocarcinoma	IV	erlotinib and ramucirumab (2016–2017)
PA12	75–80	Adenocarcinoma	IV	erlotinib (2015–2016); osimertinib (2016–2017); Chemo (2017)
PA13	60–65	Adenocarcinoma	IV	No treatment started at the time of blood collection
PA14	80–85	Adenocarcinoma	IV	taxotere/carboplatin/avastin (2015); nivolumab (2016); abraxane (2016); PD (2017)
PA15	70–75	Adenocarcinoma	IV	erlotinib (2014); rociletinib (2015–2016); osimertinib (2016–2017); rucaparib (2017); pembrolizumab (time of blood collection)
PA16	45–50	Adenocarcinoma	IV	erlotinib (2018); osimertinib (2018 to time of blood collection)
PA17	45–50	Adenocarcinoma	IIIB–IV	Untreated
PA18	65–70	Adenocarcinoma	IV	erlotinib (2012–2013); rociletinib (2013–2016); osimertinib (2016–2018); PD (2018)
PA19	60–65	Adenocarcinoma	IV	pembrolizumab (2014 to time of blood collection)
PA20	70–75	Adenocarcinoma	IV	erlotinib (2017); osimertinib (2017 to time of blood collection)
PA21	55–60	Adenocarcinoma	IV	osimertinib (2016–2017); carboplatin/pemetrexed/pembrolizumab (2017); osimertinib (2017-time of blood collection), PD (2018)
PA22	50–55	Adenocarcinoma	IV	carbo/alimta (2015); erlotinib (2015–2017); osimertinib (2017); Avastin (2017); erlotinib (2017); osimertinib (2018 to time of blood collection)
PA23	80–85	Adenocarcinoma	IV	Astellas SOLAR trial (2017); erlotinib (2017)
PA24	65–70	Mixed	IV	carbo/taxol (2017); durvalumab (2017); prednisone (2018)
HD01	35–40	Healthy		
HD02	65–70	Healthy		
HD03	40–45	Healthy		
HD04	45–50	Healthy		
HD05	35–40	Healthy		

*For EGFR assay validation, blood samples were collected from NSCLC patients (PA) and healthy donors (HD) and blinded for the user before processing. A total of 35 samples were collected, including 29 samples from 24 unique patients and 6 healthy donor samples. Patients PA03, PA04, PA05, and PA06 had serial blood draws, as indicated by the numbering.*

### Blood Sample Processing and Workflow

**FIGURE 2:**
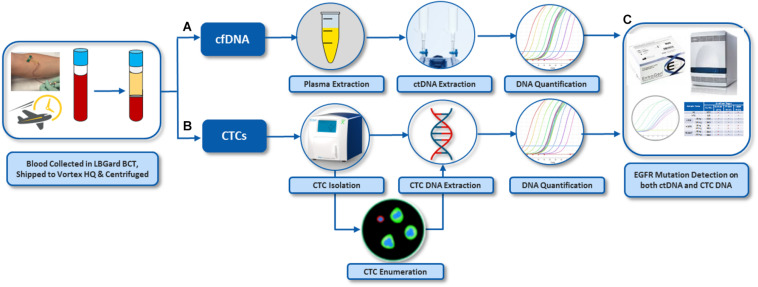
Overall workflow for *EGFR* assay on both cfDNA and CTCs from the same blood samples. **(A)** Plasma was separated from whole blood by centrifugation. cfDNA was extracted from the plasma. **(B)** CTCs were isolated from plasma-depleted blood (PDB) by VTX-1, immunostained and enumerated. DNA was also extracted from these CTCs. **(C)** *EGFR* mutation assay was performed on DNA from CTCs and plasma cfDNA. The pictures of Vortex instrument and the blood draw have been provided by Vortex Biosciences with the appropriate permission. The pictures of Qiagen reagents have been obtained from Qiagen website with the appropriate permission. Other pictures are original.

For assay validation with patient samples, blood was processed following the workflow described in Figure 2. In general, two tubes of blood were collected from each donor (healthy donors and patients), which corresponds to 16 mL of blood at the most, depending on the blood collection tube, the nurse, the donor veins, and the drawing event itself. From the two tubes of blood, the plasma was separated to isolate cfDNA. The plasma-depleted blood (PDB) was processed through Vortex technology to isolate CTCs. Plasma cfDNA and CTCs were analyzed for *EGFR* mutations.

### Plasma Separation

To separate the plasma, the blood tube was centrifuged at 1900 *g*, RT for 10 min with no brake. The plasma layer (top) was gently aspirated without disturbing the buffy coat and RBC layers underneath and transferred to a 15mL-Falcon tube. The plasma was further centrifuged at 3700 *g*, 4°C, for 15 min with slow deceleration and then transferred to a new tube and stored at −80°C until cfDNA extraction. The plasma-depleted-blood was resuspended to the original blood sample volume with 1X PBS (Gibco #20012043) and processed for CTC enrichment.

### Cancer Cell Enrichment From Blood Samples

*(i) For spiking experiments*, 2 to 4 mL of healthy whole blood or plasma-depleted blood (depending on the experiments) were diluted 10× with PBS. About 500 cells were spiked per run for “high spiking” experiments, while 50–200 cells were spiked per run for “low spiking” experiments. Cancer cells were isolated with Vortex technology, using either a manual platform described previously (23), or the VTX-1 Liquid Biopsy System (Vortex Biosciences) (20).

*(ii) For patient samples*, Isolated cells were collected into an 8-well strip for downstream fixation, immunofluorescence staining, imaging and enumeration, followed by DNA extraction and *EGFR* mutation profiling.

### Immunofluorescence Staining and Cell Enumeration

Cells enriched with Vortex technology were collected in either untreated 96 well plates (Greiner CELLSTAR^®^ #655180) or 96 Well (1 × 8 Strip Well) Clear Flat Bottom Polystyrene TC-Treated Microplates (Corning #9102). After a centrifugation (600 *g*, 1 min, RT) and aspiration of the supernatant, cells were fixed with 2% PFA (Electron Microscopy Sciences #157-4) for 10 min, permeabilized with 0.2% volume/volume Triton X-100 (Research Products International Corp) and 5% Goat Serum (Invitrogen) for 7 min, blocked with 10% Goat Serum for 30 min, and immunostained.

*(i) For the experiments assessing the different blood collection tubes*, immunostaining was performed using antibodies directed against cytokeratins (CK) (FITC, Clone CAM 5.2, BD Biosciences #347653; Clone CK3-6H5 Miltenyi Biotec #130080101), against CD45 (PE, Clone HI30, BD Pharmingen #555483) and counterstained with DAPI (Molecular Probes #D3571).

*(ii) For all EGFR spiking experiments and patient samples*, cells were stained with anti-CK FITC (Clone CAM 5.2, BD Biosciences, #347653; Clone CK3-6H5, MACS Miltenyi, #130-080-101; Clone AE1/AE3, eBioscience, #53-9003-82), anti-Vimentin AF647 (Clone V9, Abcam, #195878), anti-N-Cadherin AF647 (Clone EPR1791-4, Abcam, #195186) and anti-CD45 PE (Clone HI30, BD Biosciences, #555483) and counterstained with DAPI. H1975 cells and human WBCs were used as staining controls for all staining experiments.

The cells were imaged at 10× magnification (Axio Observer Z1, Zeiss) and enumerated using the Zen2 software (Zeiss). (i) For spike-in experiments, DAPI + /CK + /CD45- cells were identified as cancer cells while DAPI + /CK-/CD45 + cells were counted as WBCs. Capture efficiency was calculated as the number of cancer cells recovered over the total number of cancer cells spiked into the blood. Capture purity was calculated as the number of cancer cells isolated over the total number of cells collected, i.e., cancer cells and WBCs. (ii) For patient samples, putative CTCs were identified using the criteria described previously (21). Basically, potential CTCs were identified as nucleated cells (DAPI +) that are CD45- and either CK + /Vim-/ NCad-, CK + /Vim + /NCad + or CK-/Vim + /NCad + . WBCs were identified as nucleated cells (DAPI +) that are CK- and CD45 + . Cell populations were documented and the number of CTCs/mL of whole blood calculated.

For complimentary analysis, the level of cell debris can be evaluated. Following cell immunofluorescence staining, collection wells are entirely imaged with 10X magnification, both with the adequate fluorescent channels and brightfield channels. The debris are then very clearly recognizable: a qualitative and visual debris assessment can thus be defined while scanning through the wells.

### DNA Extraction and Quantification

*(i) cfDNA from plasma was extracted using* the QIAamp Circulating Nucleic Acid Kit (Qiagen #55114). Thawed plasma was lysed using buffer ACL and Proteinase K at 60°C for 30 min and then mixed with buffer ACB to enable cfDNA binding onto the column. The column was then washed and cfDNA was eluted in 50-100 μL water.

*(ii) DNA from fixed and stained cells was extracted using* QIAamp DNA Micro kit (Qiagen) with a modified protocol as described previously (27). Briefly, the well-plates were centrifuged at 250 *g* for 2 min and the supernatant from each well was carefully removed, leaving behind ∼50 μL per well. Tissue lysis buffer ATL and proteinase K were added and incubated overnight at 60°C. Then, the lysate was transferred from the well-plate to microcentrifuge tubes. Lysis buffer AL was added to help the binding of the DNA in the lysate onto the QiaAmp column. The loaded column was then washed with buffers AW1 and AW2, the bound DNA was eluted in 25 μL of water.

*(iii) DNA Quantification.* When the DNA yield was expected to be high, such as for the DNA extracted from cell lines, DNA was quantified using Qubit^TM^ 3.0 Fluorometer (Thermo Fisher) and Qubit^®^ dsDNA HS Assay Kit (Thermo Fisher). To quantify the DNA extracted from a low number of cells, a more sensitive and accurate method was needed. Therefore, an absolute quantitative PCR was performed using 7500 Fast Real-Time PCR system (Applied Biosystems^®^) and human long interspersed nuclear element-1 (hLINE-1) as the targeted gene (Forward primer: 5′-TCACTCAAAGCCGCTCAACTAC-3′ and Reverse primer: 5′-TCTGCCTTCATTTCGTTATGTACC-3′) (28). Serial dilutions of normal human genomic reference DNA (Roche Diagnostics Corporation) were used as standards.

### Multiplex qPCR-based *EGFR* Mutation Detection

*EGFR* mutation profiling was performed on cfDNA and CTC DNA samples using an ultra-sensitive multiplex qPCR-based ctEGFR kit (EntroGen), which detects L858R mutation in Exon 21, T790M mutation in Exon 20, and 48 different deletions in Exon 19. This commercial kit has been designed and validated for cfDNA with a limit of detection (LOD) of 0.4% for Ex19del (Cy5), 0.4% for T790M (FAM) and 2.5% for L858R (ROX), when using 5 ng of cfDNA as starting material. The assay was optimized in terms of sample input and assay sensitivity to be compatible with CTCs: For each sample, the 3 targeted mutations and an internal control were multiplexed in a single PCR reaction using Cy5, FAM, ROX, and VIC fluorescent probes, at a threshold set as 30,000/Cy5; 50,000/FAM; 100,000/ROX; and 20,000/VIC, with the baseline set to 3 for the first cycle and 22 for the last cycle following the manual. A positive mutation result was then determined based on the Ct criteria recommended by EntroGen.

## Results

### ctEGFR Assay Characterization for Rare Cancer Cells

**FIGURE 3:**
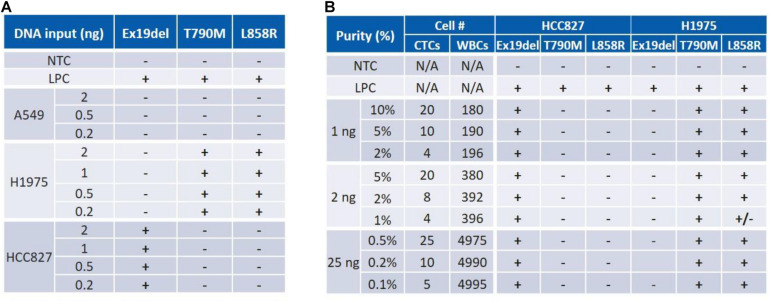
Characterization of an assay for both DNA input and sensitivity (purity) using cancer cell samples. **(A)** ctEGFR kit was tested with pure cell line DNA. Mutant DNA amount was identified for as low as 0.2 ng. **(B)** The assay sensitivity (LOD) was assessed for a range of purity varying from 0.1 to 2% for a total DNA amount varying from 25 to 1 ng. NTC, No Template Control. LPC, Low Positive Control. Expected mutations are Ex19del for HCC827; T790M and L858R for H1975.

ctEGFR kit from EntroGen is designed for cfDNA for 0.4% LOD in 5 ng of DNA input. To evaluate this assay for CTCs, 3 NSCLC cell lines (A549, H1975, HCC827) with representative *EGFR* mutations (Supplementary Figure 1) were used as surrogate to characterize the CTC workflow. *In terms of DNA input*, the corresponding *EGFR* mutation was successfully detected for each cell line for DNA quantities as low as 0.2 ng, which corresponds to approximately 33 cells (Figure 3A). *To assess the assay sensitivity*, DNA from cancer cells and WBCs were mixed at different ratios mimicking a typical Vortex output, i.e., from 1 to 25 ng of total DNA with as low as 4 CTCs among 180 to 5000 WBCs, corresponding to a purity ranging from 0.1 to 10% (Figure 3B). All *EGFR* mutations tested were successfully detected at 0.1% purity with 25 ng DNA as starting material (∼5 CTCs among 5000 WBCs) and at 2% purity with 1 ng DNA input (∼4 CTCs among 200 WBCs).

### Characterization of the cfDNA – CTC DNA Workflow

**FIGURE 4:**
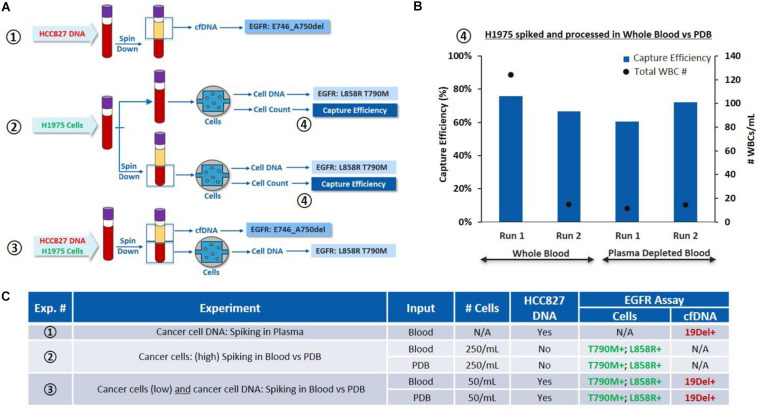
Characterization of the overall workflow with cancer cells spiked in whole blood and plasma depleted blood. **(A)** Schematic of the experiments performed: ①HCC827 DNA is spiked into whole blood (0.5 ng/2 mL of blood), cfDNA is tested for *EGFR* mutations. ②H1975 cells are spiked in whole blood (250 cells/mL of blood) and processed through VTX-1, from the whole blood or PDB. Capture efficiency and *EGFR* mutations are assessed. ③H1975 cells and HCC827 DNA are spiked in whole blood. *EGFR* mutations are evaluated on the “cfDNA” fraction and on DNA extracted from the isolated cells. **(B)** Comparison of capture performance, i.e., capture efficiency and capture purity (through WBC count), for H1975 cells spiked into whole blood and processed through VTX-1, as whole blood or as plasma depleted blood (④) (*N* = 2). **(C)** Side by side comparison of the *EGFR* mutation results obtained from experiments ①, ②, and ③. All pictures are original and courtesy of Vortex Biosciences.

*(i) On one hand, to validate the cfDNA workflow*, HCC827 DNA was spiked into EDTA whole blood at 0.5 ng per 2 mL of blood (Experiment ①, Figure 4). cfDNA was extracted from the plasma and tested for *EGFR* mutations. 19Del was successfully detected as expected.

*(ii) On the other hand, to validate the cell workflow* and evaluate the impact of plasma depletion on cell recovery, H1975 cells were spiked in whole blood at 250 cells/mL of blood and processed through VTX-1, either from the whole blood or from the plasma-depleted blood (PDB) (Experiment ②, Figure 4). Capture efficiency was similar between the whole blood (an average recovery of 70%) and the PDB (an average recovery of 66%) for *N* = 2 (Figure 4B). T790M and L858R mutations were successfully detected for both conditions, confirming that PDB can be used as an input sample for CTC isolation.

*(iii) Finally, to assess the combined workflow*, H1975 cells and HCC827 DNA were simultaneously spiked in the whole blood at the same spiking ratio (Experiment ③, Figure 4). Plasma was extracted from the blood and the PDB was processed through VTX-1 for cancer cell enrichment. *EGFR* mutations were evaluated from both the plasma cfDNA and the DNA from the cells isolated. All expected *EGFR* mutations were successfully detected, which confirmed the possibility with VTX-1 to assess both the cfDNA and the CTC DNA from the same tube of blood.

### Assessment of Blood Collection Tubes for 24 h and 48 h RT Storage

**FIGURE 5:**
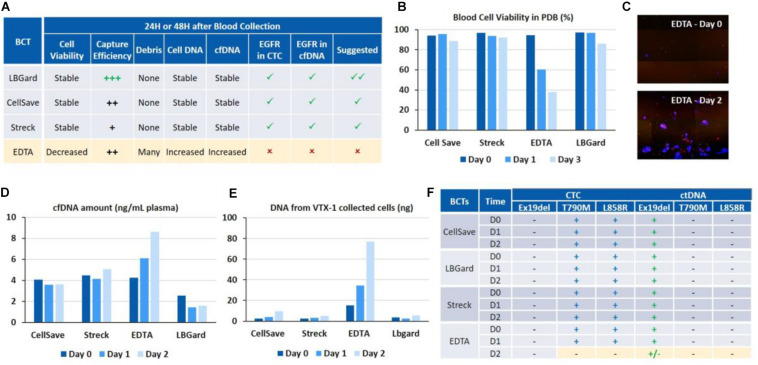
Assessment of different blood collection tubes for *EGFR* assays on cfDNA and CTC DNA after 24 and 48 h of blood storage. **(A)** Overall performance of the different BCTs. LBgard is identified as the preferred BCT as opposed to EDTA, which performance significantly worsens over the storage duration. **(B)** Initial blood cell viability in the plasma-depleted blood sample, before VTX-1 processing. **(C)** Presence of numerous DAPI + debris, i.e., nucleus from dead cells, in the VTX-1 output from EDTA BCTs after 48 h. **(D)** cfDNA yield from the plasma workflow. **(E)** Cell DNA yield from the cell workflow after VTX-1 processing. **(F)** *EGFR* mutation detection for cfDNA and CTC DNA, at Day 0, 1, and 2. All pictures are original.

Blood was collected in 4 different blood collection tubes (BCT): EDTA, CellSave, Streck ctDNA and LBgard. Around 600 H1975 cells along with 1.5 ng of HCC827 cell DNA were spiked in 12 mL of whole blood from each type of BCT, in order to have a final concentration of 100 cells and 250 pg DNA per 2 mL blood, respectively. The spiked blood from each BCT was then processed as indicated in the combined workflow described above, on Day 0 (i.e., immediately after spiking), Day 1 (24 h post-spiking) and Day 2 (48 h post-spiking). Initial blood cell viability, cell capture efficiency and purity after VTX-1 processing, debris in the VTX-1 output sample, cfDNA yield, CTC DNA yield and *EGFR* mutations were all evaluated for each of these conditions and recapitulated in Figure 5A.

#### Initial Blood Cell Viability

Blood cell viability of the plasma depleted blood was estimated for each BCT and at each time point before VTX-1 processing (Figure 5B). Cell viability (>80%) remained stable over 48 h of storage for CellSave, Streck, and LBgard. For EDTA, however, cell viability dropped significantly from 94% (Day 0) to 60% (Day 1), and to as low as 37% (Day 2).

#### VTX-1 Performance and Debris

Capture efficiency for the 4 BCTs (data not shown) spiked with MCF7 breast cancer cells indicated a worse recovery for EDTA at Day 1 and Day 2, while Streck was lower (data not available). LBGard, however, had the best capture efficiency over time, slightly higher than CellSave. Images from the VTX-1 output indicated the presence of numerous DAPI + debris enriched from the EDTA blood after a long storage time, and this phenomenon became worse over time (Figure 5C).

#### DNA Yield

cfDNA extraction yields (Figure 5D) were consistent between CellSave, Streck and LBgard BCTs for the 3 timepoints, ranging from 2 to 5 ng/mL of plasma, with LBgard showing the lowest cfDNA yield. The cfDNA yield for EDTA was the same as the 3 others BCTs at Day 0 but increased with the storage duration, from 4.25 ng/mL at Day 0 to 6.1 ng/mL at Day 1 and 8.63 ng/mL at Day 2, indicating the significant presence of non-specific DNA, probably coming from the numerous debris observed from cell lysis, usually due to WBC lysis. For the CTC DNA yield (Figure 5E), CellSave, Streck and LBgard again showed similar and stable results for Day 0 and Day 1, with a slight increase at Day 3. For EDTA, the quantity of DNA extracted from the cellular output of VTX-1 was much higher than for the other BCTs at Day 0 and, again, increased further at Day 1 and Day 2, confirming the impact of debris presence.

#### EGFR Assay Validation

The expected *EGFR* mutations could be detected from all four BCTs, from both cfDNA and CTC DNA, at Day 0 and Day 1 (Figure 5F). At Day 2, however, the mutations were missed from EDTA tubes due to the higher background of debris DNA, while they were successfully detected from CellSave, Streck, and LBgard BCTs.

Overall, LBGard tubes demonstrated the best performance in terms of DNA yield and cell recovery, answering all the needs for this combined assay and was selected for the patient samples.

### EGFR Mutation Profiling on Patient Samples

#### CTC vs. cfDNA

Blood samples from 24 metastatic NSCLC patients and six age-matched healthy donors were analyzed for *EGFR* mutations on both cfDNA and CTC DNA (Table 2). No *EGFR* mutation was detected in the cohort of healthy donors, neither in cfDNA nor in cell DNA. Among 24 patients, *EGFR* mutations were detected in 11 patients (45.8%), from either cfDNA or from CTCs. For 3/11 patients, the same mutation was identified from both cfDNA and CTCs. For 7/11 patients, the mutation was detected from cfDNA but not from CTCs, and conversely 1 sample showed an exon 19 deletion mutation only in CTCs but not in cfDNA. To confirm this last result, two additional blood samples were collected from this patient at 1 month and 5 months follow-ups, after the initial blood draw. This same exon 19 deletion was detected in CTCs after 1 month but was not detected again after 5 months.

**TABLE 2:** EGFR assay comparison.

	EGFR Assay – Vortex			EGFR Assay – UCLA			
						Concordance to Tissue – UCLA	Concordance to cfDNA – UCLA
ID	CTCs	cfDNA	CTCs + cfDNA	Tissue	cfDNA		
PA01	ND	Exon 19 del	Exon 19 del	Exon 19 del (Lung; 2015)		Yes	N/A

PA02	ND	ND	ND	Exon 19 del (Lung; 2010) T790M (Lung; 2017)		No (missed)	N/A

PA03.1	Exon 19 del	ND	Exon 19 del	ND (Lung; 2016)		No (extra)	N/A
PA03.2	Exon 19 del	ND	Exon 19 del				
PA03.3	ND	ND	ND				

PA04.1	ND	ND	ND	ND (Lung; 2016)		Yes	N/A
PA04.2	ND	ND	ND				

PA05.1	ND	Exon 19 del	Exon 19 del		Exon 19 del (Guardant360; 2016)	N/A	Yes
PA05.2	ND	Exon 19 del	Exon 19 del				

PA06.1	ND	L858R	L858R	L858R (LN; 2017)	L858R + T790M (Guardant360; 2016)	Yes	Yes
PA06.2	ND	L858R	L858R				

PA07	ND	ND	ND	Exon 19 del (Lung; 2016)	ND (Guardant360; 2017)	No (missed)	Yes

PA08	Exon 19 del	Exon 19 del	Exon 19 del	Exon 19 del (Lung; 2010) T790M (Lung; 2015) ND (Pleural Fluid; 2017);	ND (Guardant360; 2017)	Yes / No	No (extra)

PA09	ND	L858R	L858R	L858R(LN; 2017)		Yes	N/A

PA10	ND	ND	ND	L858R (Lung; 2017)		No (missed)	N/A

PA11	ND	L858R	L858R	L858R (Lung; 2016)		Yes	N/A

PA12	L858R + T790M	L858R + T790M	L858R + T790M	L858R (LN; 2015)	L858R + T790M (Biocept; 2015)	Yes	Yes

PA13	ND	L858R	L858R	L858R (Lung)		Yes	N/A N/A

PA14	ND	ND	ND	ND(LN;2016)		Yes	

PA15	ND	ND	ND	L858R + T790M(LN;2014)		No (missed)	N/A N/A

PA16	ND	ND	ND	Exon 19 del (Lung; 2017)		No (missed)	

PA17	ND	ND	ND	ND (Lung; 2017)		Yes	N/A

PA18	ND	Exon 19 del	Exon 19 del	Exon 19 del (Lung; 2012)	Exon 19 del + T790M (Guardant; 2016); Exon 19 del (Guardant; 2017)	Yes	Yes

PA19	ND	ND	ND	L858R (Lung; 2017)	No (missed)	N/A	

PA20	ND	ND	ND	L858R (Lung; 2017); L858R + T790M (Pleural Fluid; 2017)	L858R (Guardant360; 2017);	No (missed)	No (missed)

PA21	T790M	L858R + T790M	L858R + T790M	L858R + T790M (Lung; 2016);	L858R + T790M (Guardant360; 2017)	Yes	Yes

PA22	ND	ND	ND	Exon 19 del + T790M (Lung; 2018)	Exon 19 del + T790M (Guardant360; 2018)	No (missed)	No (missed)

PA23	ND	ND	ND	L858R (LN; 2017)		No (missed)	N/A

PA24	ND	ND	ND	ND (Lung; 2017)		Yes	N/A

HDOl	ND			N/A	N/A	N/A	
HD02							
HD03							
HD04							
HD05							
HD06							

*Analysis of 36 samples from NSCLC patients (PA) and healthy donors (HD). Side-by-side comparison of EGFR results from assays performed at UCLA and Vortex Biosciences. ND, non detected. N/A, not available. LN, Lymph Nodes.*

#### CTC and cfDNA vs. Tumor Tissue

Out of 24 patients, 13 had a concordant mutation status between tissue and combined CTC and cfDNA liquid biopsies, 10 had discordance between tissue and liquid biopsies, with one patient having more mutations detected in the liquid biopsy than in the tissue. For most of the patients, however, the tumor biopsies were analyzed up to 7 years before the blood sampling (>1 year for 12/24 patients), which might explain the discrepancy observed. Tissue biopsy was not available for 1/24 patients.

#### CTCs and cfDNA vs. UCLA Standard cfDNA Assay

Among these 24 patients, 9 had a cfDNA *EGFR* mutation analysis performed previously at a UCLA clinic (the assay used for these 9 patients is described in Table 2). Of those, 6 returned the same mutation profile as the Vortex-EntroGen combined workflow. For the 3 discordant patient samples, 1 had more mutations detected by the combined Vortex workflow.

### CTC Immunostaining and Enumeration

**FIGURE 6:**
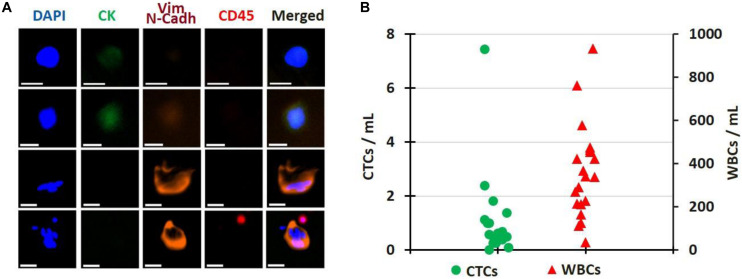
CTC Enumeration. **(A)** Gallery of CTCs, single or clustered, isolated from patient samples. Scale bar: 20 μm. **(B)** Cell enumeration per mL of blood processed for 20 patient samples; CTCs (green circles) and WBCs (red triangles). All pictures are original.

For 20 patient samples, extra blood volume was processed specifically for immunostaining and enumeration, as defined in the Methods section. Representative images of CTCs are presented in Figure 6A, while the enumeration results are plotted in Figure 6B. Patients had from 0 to 7.4 CTCs/mL of blood processed (average: 1.1 CTCs/mL, median: 0.5 CTCs/mL), with 35 to 932 WBCs/mL (average: 360.9 WBCs/mL, median: 339.8 WBCs/mL). The patient sample with the most CTCs corresponds to the first draw of Patient PA03, for which Exon 19 deletion mutation was detected only in the CTCs. No CTC enumeration was performed on healthy donor’s blood in this current study but in others (21, 25).

## Discussion

The discovery of oncogenic driver mutations of the *EGFR* gene and the approval of *EGFR* inhibitors have revolutionized the targeted individualized treatment approach in non-small-cell lung cancer (NSCLC) (29). Patients with *EGFR* exon 19 deletions or exon 21 L858R mutations are eligible for treatment with gefitinib, afatinib, erlotinib, dacomitinib and osimertinib. Patients who later develop *EGFR* T790M mutation and whose disease is progressing on or after erlotinib, gefitinib, afatinib or dacomitinib can benefit from treatment with osimertinib. *EGFR* mutation profiling and corresponding *EGFR* inhibitors have already significantly prolonged many patients’ lives. Most of the FDA approved *EGFR* mutation detection tests rely on tissue biopsies. In parallel, some plasma-based assays, such as the cobas^®^*EGFR* Mutation Test from Roche, offer a non-invasive alternative for those patients not eligible for a tissue biopsy. Liquid biopsies allow the clinicians to monitor treatment effectiveness and disease progression over time. While cfDNA *EGFR* mutation tests are widely adopted for selecting the appropriate *EGFR* inhibitor for a given patient, CTCs are less popular and not used in the clinic. No FDA approved *EGFR* mutation test is approved for CTCs. This can be explained by two reasons: First, CTCs are rare, and retrieving CTCs from a blood sample with millions of WBCs and billions of RBCs in the background is not as straightforward as separating the plasma from the blood. Second, it also requires a very good CTC enrichment system that can recover sufficient CTCs with few normal blood cells to satisfy the requirements of the downstream *EGFR* assay in terms of sensitivity and specificity.

We examined the performance of the kits available for cfDNA and selected the ones that could be compatible with CTCs as well^1^. qPCR-based assays were favored over Next-Generation Sequencing methods as they require less input DNA (e.g., as low as 2 ng) and are more sensitive (with limit of detection/LOD as low as 0.4%). Among these qPCR-based kits, the Roche cobas^®^ *EGFR* real-time PCR test targets a panel of 42 mutations in exons 18, 19, 20, and 21 but requires an amount of starting material that cannot be obtained from CTC samples (50 ng of DNA per reaction in 8 reactions^2^). Among all the *EGFR* mutations considered, only a few are clinically actionable; with the most famous examples being *EGFR* exon 19 deletions, L858R and T790M. Therefore, the ctEGFR Mutation Detection Kit from EntroGen was selected owing to its ability to simultaneously detect these 3 key *EGFR* mutations in plasma cfDNA through FAM, VIC, ROX, and CY5 fluorescent probes with very high sensitivity. This assay requires as little as 2 μg of DNA, for a LOD that can reach 0.4%. This kit was validated with cell lines mimicking a typical CTC sample in terms of DNA input and purity. Interestingly, during our research, we found a SNP c.2361 G > A (p.Q878Q) in the three cancer cell lines considered, 8 nucleotides upstream of c. 2369 C > T (p.T790M) (Supplementary Figure 1). This was not considered in the kit we started with and might affect the probes/primers and overall performance. This information was reported to EntroGen; the commercial kits revised accordingly and used for the next steps of our studies for both plasma and CTC samples. Also, the C797 mutations, osimertinib emergent, were not considered by this combined assay and could be considered later on.

Among the CTC isolation platforms currently available, some rely on different cell surface markers expressed by the cells at their surface, such as EpCAM or CK. Such platforms may not capture the CTCs lacking the markers targeted (30–32). Other platforms rely on the cell dimensions, using filtering features either in a paper filter or in a microfluidic chip. Such platforms often leave CTCs trapped on a filter or inside a chip, making their intact retrieval a challenge (33). Vortex has developed a microfluidic label-free and automated CTC isolation system, which captures cells using vortices and inertial forces (20, 22). Cells are collected in suspension in the container of choice and remain intact, which guarantees the integrity of CTC DNA or RNA signatures, for an ideal compatibility with downstream gene mutation or gene expression assays (25, 27, 34). More importantly for this study, the very little number of WBCs collected (<100 WBCs/mL blood) advantageously provides a CTC sample with a very high purity that can be then directly used for *EGFR* mutation detection (23).

One purpose of the study was to evaluate the feasibility to process and isolate CTCs from plasma depleted blood with Vortex technology. Indeed, the possibility to use the same blood sample to isolate and analyze cfDNA, extracellular vesicles, and CTCs would ensure no genomic and transcriptomic information is missed, yielding a total liquid biopsy. Collecting multiple blood samples from metastatic patients can prove difficult at times, because of the patient’s poor general condition and the blood drawn at a given clinic appointment is generally split over numerous tests. Achieving one integrated workflow to minimize the blood volume needed and sample-to-sample bias while getting the complete transcriptomic and genomic information would be both beneficial to the patient and informative to the clinician. As confirmed in our results, the capture efficiency from the whole blood and the plasma-depleted blood were similar, demonstrating that Vortex technology could work on both whole blood and plasma depleted blood. Beyond *EGFR* mutation profiling, this gives researchers the opportunity to perform more assays with one tube of blood, for example to look at exosome plasma RNA versus CTC RNA. Even more, besides plasma vs. cellular phase, future studies will consider assays on both CTCs and leukocytes, as leukocytes can be collected intact from VTX-1 waste streams.

More generally, we are targeting assays that could be used by multiple sites, i.e., assuming patient samples need to be shipped from a sampling site to a processing lab, which potentially can take up to 48 h. In this purpose, different blood collection tubes were considered and compared to the classic BD vacutainer K_2_ EDTA tube (without any preservative added) over 2 days, considering all the key steps of the combined workflow: blood viability, cfDNA amount, cell recovery, cell DNA amount, and *EGFR* mutations. We selected blood collection tubes commercially available and marketed for cfDNA and/or CTCs, such as CellSave (recommended for CTCs analysis by CellSearch), Streck cfDNA (recommended for cfDNA and CTCs), and LBgard (recommended for cfDNA and CTCs). Other blood collection tubes may have been launched on the market since, which would be interesting to consider as well moving forward. The results demonstrated that while collecting blood in classic BD vacutainer K_2_ EDTA tubes works fine for same day processing and analysis of both cfDNA and CTCs, performance was affected as soon as 24 h post draw: after processing, lots of debris was present in the blood, increasing dramatically the overall contaminating background thus the cfDNA and cell DNA amount. CellSave, Streck and LBgard tubes showed better performance, with LBgard significantly better for all the parameters evaluated. This study highlights the crucial impact of pre-analytical variables; the blood collection method and storage duration for this specific assay, which can actually apply to all liquid biopsy related assays. Ideally, even if time and resource consuming, such side-by-side assessment should be commonly performed as early as possible in the development process, considering also the storage temperature and the shipping technique.

Our results indicate that *EGFR* mutations were identified in 11 out of 24 (45.8%) patient samples tested. Among the 11 patients with *EGFR* mutations; seven mutations were identified in the cfDNA but not in the CTCs, 1 exon 19 deletion was found only in the CTCs and not in the cfDNA, while three harbored the same mutations in both cfDNA and CTC fractions. No *EGFR* mutations were detected in the healthy donor samples using our integrated workflow. Despite the relatively small patient cohort, these results provide a preliminary indication of the efficiency and specificity of our “Total Liquid Biopsy” workflow. Mutational profiles from cfDNA and gDNA from CTCs differ significantly and together may give a more comprehensive picture. These results show that the combination of cfDNA and CTCs may be more useful than either test alone. The patient who had positive CTCs but negative cfDNA had 2 subsequent blood draws within 5 months; the same exon 19 deletion was detected in the second blood draw but not in the 3rd one. Even if rare, such occurrence could still give some useful information as a complement to cfDNA alone. The overall performance of cfDNA is superior than CTCs for this DNA based assay. This might be explained by several reasons: 1/Most of the patients in this small cohort had already received chemotherapy or *EGFR* inhibitors at the time of the blood collection. A portion of CTCs might have died during the treatments, which is also indicated by the number of CTCs that is lower than in other studies (21). 2/In the case of CTC apoptosis, CTCs die and more ctDNA is released into the plasma, which increases the ctDNA portion in the cfDNA and results in better performance for ctDNA than CTCs. However, this explanation implies that new mutations would be first detected in the CTCs while cfDNA would provide a snapshot of dying cancer cells instead. Combining *EGFR* mutation analysis of both cfDNA and CTC DNA would thus be of special interest to detect earlier a new mutation and adjust accordingly the treatment regimen of NSCLC patients, for example to switch a patient to Osimertinib if T790M mutation is being detected on erlotinib, gefitinib, afatinib or dacomitinib. This point has been further described elsewhere and emphasizes the clinical importance of CTCs as a point of access to intact cancer DNA, RNA or proteins (35–37). In the study from Sundaresan et al., cfDNA or CTC analysis alone had less sensitivity vs. combining both, with a genotyping of 70 and 80% for CTCs and cfDNA, respectively, but 100% when combined (35).

In parallel, we compared the mutational profiles from cfDNA and gDNA from CTCs to the ones obtained from tissue biopsies (tumor or LN). Still, 13 out of 24 patient samples had the same mutation results for tissue vs. combined CTC + cfDNA, for more than 50% concordance. Yet, the comparison with tissue biopsies is to be taken cautiously, with an expected discrepancy, as some biopsies were analyzed up to 7 years before the blood sampling, and the patient cohort is limited. This also emphasizes the change of mutations over the course of the disease and treatment regimens, and the crucial relevance of liquid biopsies to monitor patient progression. We envision a larger study, considering a larger patient cohort with more stringent patient selection criteria, to further assess the accuracy of the assay and to enable a comparison between total liquid biopsy and tissue biopsy results.

Lastly, when we compare the Vortex combined *EGFR* cfDNA CTC workflow with some commercial assays (Guardant Health or Ion Torrent) performed at UCLA on nine cfDNA samples; there was concordance for six samples (66.7%). For two samples, commercial assays detected more mutations. For one sample, our combined workflow found more comprehensive results. These differences could be explained by different blood collection timepoints, the small patient cohort and a different sensitivity for each assay. As there is no gold standard assay, this remains unclear which is correct for each of the samples.

## Conclusion

Although the present study is limited by the small patient cohort considered, and the time gap between the tissue biopsy and the blood collection, these preliminary results present, characterize and validate a combined workflow for *EGFR* mutation analysis on cfDNA and CTCs from a single tube of blood. DNA mutation detection of a small targeted panel using qPCR is easier from cfDNA, but combining and comparing cfDNA with CTC DNA is possible with a streamlined workflow. cfDNA being potentially indicative of dying cells after therapy while CTCs living after therapy may have more valuable information, a combined workflow could provide complementary indication on the patient resistance as a “total liquid biopsy.” An extended study should be considered on a larger patient cohort, using isolated CTCs to detect *EGFR* mutations alongside with a potent heterogeneity analysis of somatic copy number alterations and mutations.

## Data Availability Statement

All datasets presented in this study are included in the article/Supplementary Material. The authors can be contacted for further information.

## Ethics Statement

The studies involving human participants were reviewed and approved by UCLA IRB 11-001798 and STANFORD IRB 5630. The patients/participants provided their written informed consent to participate in this study.

## Author Contributions

HL, MV, CW, CR, MC, CL, JCh, and ES designed the experiments. HL, MV, CW, CR, MC, and CL performed the experiments. MM, JCa, VH, SSJ, EG, and JG identified the donors and/or obtained the blood samples. HL, MV, CW, CR, JCh, and ES analyzed the results. HL, MV, CR, and ES wrote the manuscript with assistance from all authors. All authors have read and approved this manuscript.

## Conflict of Interest

HL, MV, CW, CR, MC, CL, JCh, SC, DDC, and ES have financial interests in Vortex Biosciences. JCh, DDC, and ES have interests in the intellectual property described herein for Vortex Biosciences technology. UCLA acknowledges research funding support from Vortex Biosciences (MM, JCa, and DDC). The remaining authors declare that the research was conducted in the absence of any commercial or financial relationships that could be construed as a potential conflict of interest.

## Supplementary Material

Supplementary Figure S1 NSCLC cell line characterization. **(A)** 3 human NSCLC cell lines with known *EGFR* mutations – A549 (ATCC^®^ CCL-185^TM^, wild type), H1975 (ATCC^®^ CRL-5908^TM^, T790M and L858R mutations), and HCC827 (ATCC^®^ CRL-2868^TM^, 19 deletion) – were used to characterize the CTC workflow. For each cell line, cell mutations were confirmed by Sanger sequencing. **(B)** The amplified PCR products corresponding to the *EGFR* exons 19, 20, 21 covering 19 deletion, T790M and L858R mutations were verified by E-Gel Electrophoresis and subjected to Sanger sequencing. **(C)** The sequencing results demonstrate the presence of the expected mutations in the corresponding cell lines. All the pictures are original.

Supplementary Figure S2 Primer sequences. The primers were specifically designed to amplify the regions of the *EGFR* exons 19, 20, and 21 covering 19 deletion, T790M and L858R mutations. For all spiking experiments, cells at a confluency of 40–60% were dissociated using TrypLE express (Gibco). The concentration of live cells, cell viability and average cell diameter were measured using an automated cell counter (Cellometer K2, Nexcelom) after staining with Acridine Orange and Propidium Iodide (AOPI) dyes. Stock cell suspensions were serially diluted in complete medium to obtain the desired final concentrations before spiking in blood.

## References

[1] Siegel RL Miller KD Jemal A. Cancer statistics. *CA Cancer J Clin.* (2020) 70:7–30. 10.3322/caac.21590 31912902

[2] Crinò L Weder W van Meerbeeck J Felip E ESMO Guidelines Working Group Early stage and locally advanced (non-metastatic) non-small-cell lung cancer: ESMO clinical practice guidelines for diagnosis, treatment and follow-up. *Ann Oncol Suppl.* (2010) 5:v103–15. 10.1093/annonc/mdq207 20555058

[3] Midha A Dearden S McCormack R. EGFR mutation incidence in non-small-cell lung cancer of adenocarcinoma histology: a systematic review and global map by ethnicity (mutMapII). *Am J Cancer Res.* (2015) 5:2892–911. 26609494 PMC4633915

[4] Gelatti ACZ Drilon A Santini FC. Optimizing the sequencing of tyrosine kinase inhibitors (TKIs) in epidermal growth factor receptor (EGFR) mutation-positive non-small cell lung cancer (NSCLC). *Lung Cancer.* (2019) 137:113–122. 10.1016/j.lungcan.2019.09.017 31568888 PMC7478849

[5] Bethune G Bethune D Ridgway N Xu Z. Epidermal growth factor receptor (EGFR) in lung cancer: an overview and update. *J Thorac Dis.* (2010) 2:48–51. 22263017 PMC3256436

[6] Nakagawa K Garon EB Seto T Nishio M Ponce Aix S Paz-Ares L Ramucirumab plus erlotinib in patients with untreated, EGFR-mutated, advanced non-small-cell lung cancer (RELAY): a randomised, double-blind, placebo-controlled, phase 3 trial. *Lancet Oncol.* (2019) 20:1655–69. 10.1016/S1470-2045(19)30634-5 31591063

[7] Hosomi Y Morita S Sugawara S Kato T Fukuhara T Gemma A Gefitinib alone versus Gefitinib plus chemotherapy for non–small-cell lung cancer with mutated epidermal growth factor receptor: NEJ009 study. *J Clin Oncol.* (2020) 38:115–23. 10.1200/JCO.19.0148 31682542

[8] Noronha V Maruti Patil V Joshi A Menon N Chougule A Mahajan A Gefitinib versus Gefitinib plus pemetrexed and carboplatin chemotherapy in EGFR-mutated lung cancer. *J Clin Oncol.* (2020) 38:124–36. 10.1200/JCO.19.01154 31411950

[9] Ramalingam SS Vansteenkiste J Planchard D Cho BC Gray JE Ohe Y Overall survival with Osimertinib in untreated, EGFR-mutated advanced NSCLC. *N Engl J Med.* (2020) 382:41–50. 10.1056/NEJMoa1913662 31751012

[10] Marrugo-Ramírez J Mir M Samitier J. Blood-based cancer biomarkers in liquid biopsy: a promising non-invasive alternative to tissue biopsy. *Int J Mol Sci.* (2018) 19:2877. 10.3390/ijms19102877 30248975 PMC6213360

[11] Arneth B. Update on the types and usage of liquid biopsies in the clinical setting: a systematic review. *BMC Cancer.* (2018) 18:527. 10.1186/s12885-018-4433-3 29728089 PMC5935950

[12] Calabuig-Fariñas S Jantus-Lewintre E Herreros-Pomares A Camps C. Circulating tumor cells versus circulating tumor DNA in lung cancer—which one will win? *Transl Lung Cancer Res.* (2016) 5:466–82. 10.21037/tlcr.2016.10.02 27826528 PMC5099512

[13] Singh AP Li S Cheng H. Circulating DNA in EGFR-mutated lung cancer. *Ann Transl Med.* (2017) 5:379. 10.21037/atm.2017.07.10 29057239 PMC5635258

[14] Mason J Blyth B MacManus MP Martin OA. Treatment for non-small-cell lung cancer and circulating tumor cells. *Lung Cancer Manag.* (2018) 6:129–39. 10.2217/lmt-2017-0019 30643579 PMC6310303

[15] Kapeleris J Kulasinghe A Warkiani ME Vela I Kenny L O’Byrne K The prognostic role of circulating tumor cells (CTCs) in lung cancer. *Front Oncol.* (2018) 8:311. 10.3389/fonc.2018.00311 30155443 PMC6102369

[16] Santarpia M Liguori A D’Aveni A Karachaliou N Gonzalez-Cao M Daffinà MG Liquid biopsy for lung cancer early detection. *J Thorac Dis.* (2018) 10 (Suppl. 7):S882–97. 10.21037/jtd.2018.03.81 29780635 PMC5945693

[17] Phallen J Leal A Woodward BD Forde PM Naidoo J Marrone KA Early noninvasive detection of response to targeted therapy in non-small cell lung cancer. *Cancer Res.* (2019) 79:1204–13. 10.1158/0008-5472.CAN-18-1082 30573519 PMC6481620

[18] Anagnostou V Forde PM White JR Niknafs N Hruban C Naidoo J Dynamics of tumor and immune responses during immune checkpoint blockade in non-small cell lung cancer. *Cancer Res.* (2019) 79:1214–25. 10.1158/0008-5472.CAN-18-1127 30541742 PMC6432636

[19] Liang H Huang J Wang B Liu Z He J Liang W. The role of liquid biopsy in predicting post-operative recurrence of non-small cell lung cancer. *J Thorac Dis.* (2018) 10 (Suppl. 7):S838–45. 10.21037/jtd.2018.04.08 29780630 PMC5945696

[20] Lemaire CA Liu SZ Wilkerson CL Ramani VC Barzanian NA Huang KW Fast and label-free isolation of circulating tumor cells from blood: from a research microfluidic platform to an automated fluidic instrument, VTX-1 liquid biopsy system. *SLAS Technol.* (2018) 23:16–29. 10.1177/2472630317738698 29355087

[21] Che J Yu V Dhar M Renier C Matsumoto M Heirich K Classification of large circulating tumor cells isolated with ultra-high throughput microfluidic Vortex technology. *Oncotarget.* (2016) 7:12748–60. 10.18632/oncotarget.7220 26863573 PMC4914319

[22] Sollier E Go DE Che J Gossett DR O’Byrne S Weaver WM Size-selective collection of circulating tumor cells using Vortex technology. *Lab Chip.* (2014) 14:63–77. 10.1039/C3LC50689D 24061411

[23] Kidess-Sigal E Liu HE Triboulet MM Che J Ramani VC Visser BC Enumeration and targeted analysis of KRAS, BRAF and PIK3CA mutations in CTCs captured by a label-free platform: comparison to cfDNA and tissue in metastatic colorectal cancer. *Oncotarget.* (2016) 7:85349–64. 10.18632/oncotarget.13350 27863403 PMC5356741

[24] Sinkala E Sollier-Christen E Renier C Rosàs-Canyelles E Che J Heirich K Profiling protein expression in circulating tumour cells using microfluidic western blotting. *Nat Commun.* (2017) 8:14622. 10.1038/ncomms14622 28332571 PMC5376644

[25] Renier C Pao E Che J Liu HE Lemaire CA Matsumoto M Label-free isolation of prostate circulating tumor cells using Vortex microfluidic technology. *NPJ Precis Oncol.* (2017) 2017:15. 10.1038/s41698-017-0015-0 PMC5859469 29872702

[26] Dhar M Wong J Che J Matsumoto M Grogan T Elashoff D Evaluation of PD-L1 expression on vortex-isolated circulating tumor cells in metastatic lung cancer. *Sci Rep.* (2018) 8:2592. 10.1038/s41598-018-19245-w 29416054 PMC5803213

[27] Liu HE Triboulet M Zia A Vuppalapaty M Kidess-Sigal E Coller J Workflow optimization of whole genome amplification and targeted panel sequencing for CTC mutation detection. *NPJ Genom Med.* (2017) 2:34. 10.1038/s41525-017-0034-3 PMC5677973 29263843

[28] Rago C Huso DL Diehl F Karim B Liu G Papadopoulos N Serial assessment of human tumor burdens in mice by the analysis of circulating DNA. *Cancer Res.* (2007) 67:9364–70. 10.1158/0008-5472.CAN-07-0605 17909045

[29] Singh N Bal A Aggarwal AN Das A Behera D. Clinical outcomes in non-small-cell lung cancer in relation to expression of predictive and prognostic biomarkers. *Future Oncol.* (2010) 6:741–67. 10.2217/fon.10.30 20465389

[30] Ramalingam N Jeffrey SS. Future of liquid biopsies with growing technological and bioinformatics studies: opportunities and challenges in discovering tumor heterogeneity with single-cell level analysis. *Cancer J.* (2018) 24:104–108. 10.1097/PPO.0000000000000308 29601337 PMC5880298

[31] Murlidhar V Rivera-Báez L Nagrath S. Affinity versus label-free isolation of circulating tumor cells: who wins? *Small.* (2016) 12:4450–4463. 10.1002/smll.201601394 27436104

[32] Lampignano R Schneck H Neumann M Fehm T Neubauer H. Enrichment, isolation and molecular characterization of EpCAM-negative circulating tumor cells. *Adv Exp Med Biol.* (2017) 994:181–203. 10.1007/978-3-319-55947-6_10 28560675

[33] Mu Z Benali-Furet N Uzan G Znaty A Ye Z Paolillo C Detection and characterization of circulating tumor associated cells in metastatic breast cancer. *Int J Mol Sci.* (2016) 17:1665. 10.3390/ijms17101665 27706044 PMC5085698

[34] Ju JA Lee CJ Thompson KN Ory EC Lee RM Mathias TJ Partial thermal imidization of polyelectrolyte multilayer cell tethering surfaces (TetherChip) enables efficient cell capture and microtentacle fixation for circulating tumor cell analysis. *Lab Chip.* (2020) 20:2872–88. 32744284 10.1039/d0lc00207k PMC7595763

[35] Sundaresan TK Sequist LV Heymach JV Riely GJ Jänne PA Koch WH Detection of T790M, the acquired resistance EGFR mutation, by tumor biopsy versus noninvasive blood-based analyses. *Clin Cancer Res.* (2016) 22:1103–10. 10.1158/1078-0432.CCR-15-1031 26446944 PMC4775471

[36] Keup C Storbeck M Hauch S Hahn P Sprenger-Haussels M Tewes M Cell-free DNA variant sequencing using CTC-depleted blood for comprehensive liquid biopsy testing in metastatic breast cancer. *Cancers (Basel).* (2019) 11:238. 10.3390/cancers11020238 30781720 PMC6406821

[37] Keup C Storbeck M Hauch S Hahn P Sprenger-Haussels M Hoffmann O Multimodal targeted deep sequencing of circulating tumor cells and matched cell-free DNA provides a more comprehensive tool to identify therapeutic targets in metastatic breast cancer patients. *Cancers (Basel).* (2020) 12:1084. 10.3390/cancers12051084 32349306 PMC7281124

