# Supplementary material for: Detection of EGFR Mutations in cfDNA and CTCs, and Comparison to Tumor Tissue in Non-Small-Cell-Lung-Cancer (NSCLC) Patients
Source: Front Oncol. 2020 Oct 8;10:572895. doi: 10.3389/fonc.2020.572895 (PMC7578230; doi:10.3389/fonc.2020.572895)
Supplement: Supplementary Figure S1 — NSCLC cell line characterization. (A) 3 human NSCLC cell lines with known EGFR mutations – A549 (ATCC® CCL-185TM, wild type), H1975 (ATCC® CRL-5908TM, T790M and L858R mutations), and HCC827 (ATCC® CRL-2868TM, 19 deletion) – were used to characterize the CTC workflow. For each cell line, cell mutations were confirmed by Sanger sequencing. (B) The amplified PCR products corresponding to the EGFR exons 19, 20, 21 covering 19 deletion, T790M and L858R mutations were verified by E-Gel Electrophoresis and subjected to Sanger sequencing. (C) The sequencing results demonstrate the presence of the expected mutations in the corresponding cell lines. All the pictures are original. [file Data_Sheet_1.docx]

**Supplementary Information**

**Detection of EGFR Mutations in cfDNA and CTCs, and comparison to Tumor Tissue in Non-Small-Cell-Lung-Cancer (NSCLC) Patients**


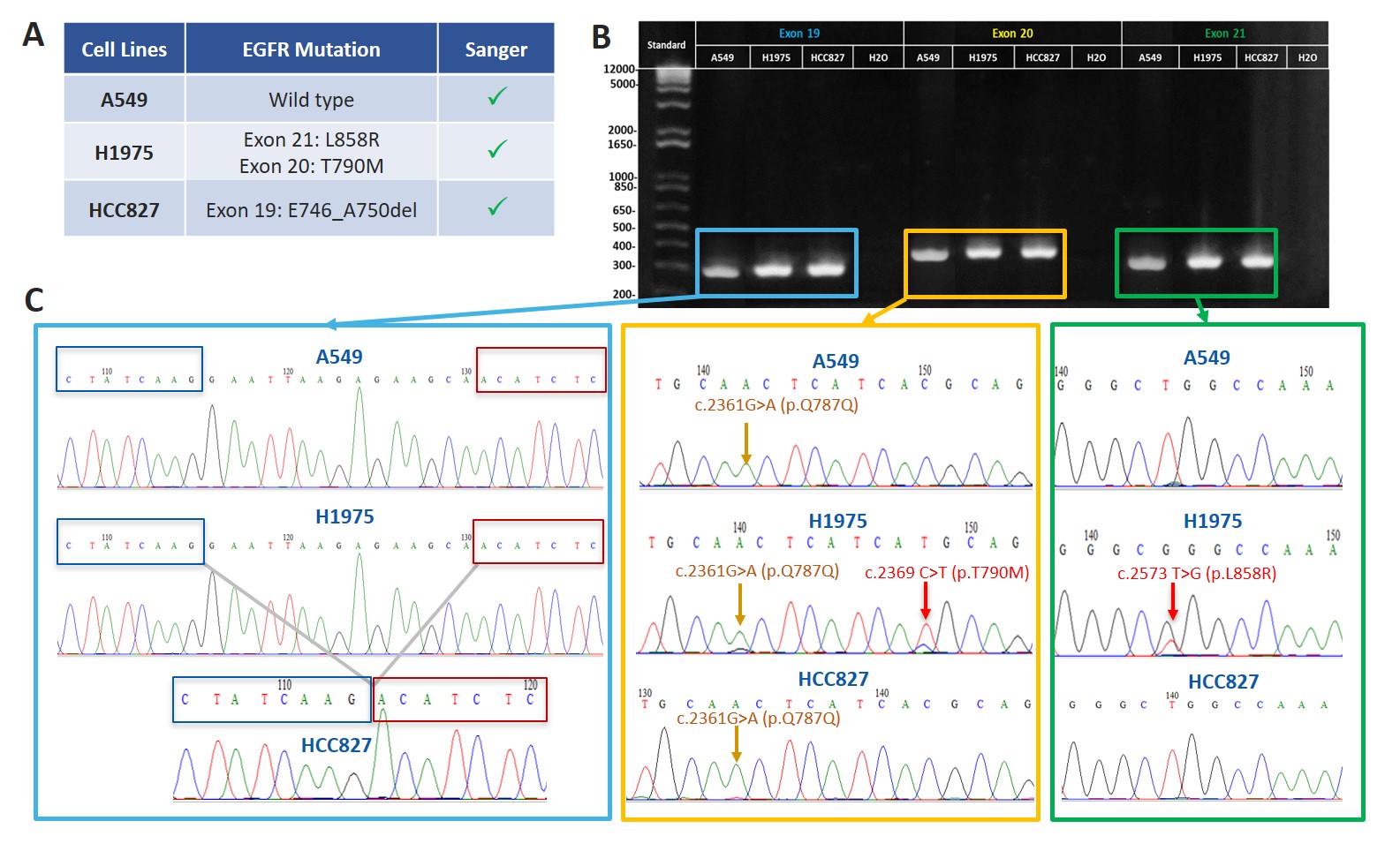
 **Supp. Figure 1: NSCLC cell line characterization.** (A) 3 human NSCLC cell lines with known *EGFR* mutations - A549 (ATCC® CCL-185™, wild type), H1975 (ATCC® CRL-5908™, T790M and L858R mutations), and HCC827 (ATCC® CRL-2868™, 19 deletion) - were used to characterize the CTC workflow. For each cell line, cell mutations were confirmed by Sanger sequencing. (B) The amplified PCR products corresponding to the *EGFR* exons 19, 20, 21 covering 19 deletion, T790M and L858R mutations were verified by E-Gel Electrophoresis and subjected to Sanger sequencing. (C) The sequencing results demonstrate the presence of the expected mutations in the corresponding cell lines. All the pictures are original.


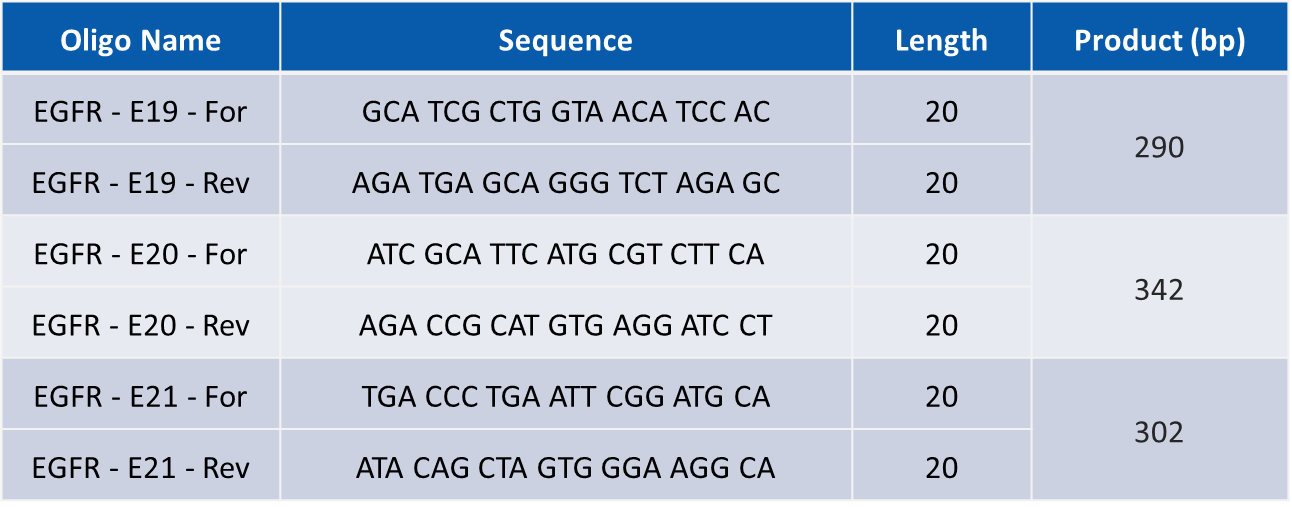


**Supp. Figure 2: Primer sequences.** The primers were specifically designed to amplify the regions of the *EGFR* exons 19, 20, and 21 covering 19 deletion, T790M and L858R mutations.
